# Genetic architecture underlying the expression of eight α-amylase trypsin inhibitors

**DOI:** 10.1007/s00122-021-03906-y

**Published:** 2021-07-10

**Authors:** Khaoula EL Hassouni, Malte Sielaff, Valentina Curella, Manjusha Neerukonda, Willmar Leiser, Tobias Würschum, Detlef Schuppan, Stefan Tenzer, C. Friedrich H. Longin

**Affiliations:** 1 State Plant Breeding Institute, University of Hohenheim, Fruwirthstr. 21, 70599 Stuttgart, Germany; 2 Institute for Immunology, University Medical Center of the Johannes Gutenberg-University Mainz, Langenbeckstr. 1, 55131 Mainz, Germany; 3 Institute of Translational Immunology and Research Center for Immune Therapy, University Medical Center of the Johannes Gutenberg-University Mainz, Langenbeckstr. 1, 55131 Mainz, Germany; 4 Institute of Plant Breeding, Seed Science and Population Genetics, University of Hohenheim, Fruwirthstr. 21, 70599 Stuttgart, Germany; 5 Division of Gastroenterology, Beth Israel Deaconess Medical Center, Harvard Medical School, 330 Brookline Ave, Boston, MA 02215 USA

## Abstract

**Key message:**

Wheat cultivars largely differ in the content and composition of ATI proteins, but heritability was quite low for six out of eight ATIs. The genetic architecture of ATI proteins is built up of few major and numerous small effect QTL.

**Abstract:**

Amylase trypsin inhibitors (ATIs) are important allergens in baker’s asthma and suspected triggers of non-celiac wheat sensitivity (NCWS) inducing intestinal and extra-intestinal inflammation. As studies on the expression and genetic architecture of ATI proteins in wheat are lacking, we evaluated 149 European old and modern bread wheat cultivars grown at three different field locations for their content of eight ATI proteins. Large differences in the content and composition of ATIs in the different cultivars were identified ranging from 3.76 pmol for ATI CM2 to 80.4 pmol for ATI 0.19, with up to 2.5-fold variation in CM-type and up to sixfold variation in mono/dimeric ATIs. Generally, heritability estimates were low except for ATI 0.28 and ATI CM2. ATI protein content showed a low correlation with quality traits commonly analyzed in wheat breeding. Similarly, no trends were found regarding ATI content in wheat cultivars originating from numerous countries and decades of breeding history. Genome-wide association mapping revealed a complex genetic architecture built of many small, few medium and two major quantitative trait loci (QTL). The major QTL were located on chromosomes 3B for ATI 0.19-like and 6B for ATI 0.28, explaining 70.6 and 68.7% of the genotypic variance, respectively. Within close physical proximity to the medium and major QTL, we identified eight potential candidate genes on the wheat reference genome encoding structurally related lipid transfer proteins. Consequently, selection and breeding of wheat cultivars with low ATI protein amounts appear difficult requiring other strategies to reduce ATI content in wheat products.

**Supplementary Information:**

The online version contains supplementary material available at 10.1007/s00122-021-03906-y.

## Introduction

Wheat (*Triticum aestivum* subsp*. aestivum*) is one of the predominant crops globally and the most consumed cereal worldwide (FAO 2019) providing a high nutritional value with fibers, minerals, vitamins, proteins and starch (Shewry 2009). However, wheat consumption can cause adverse health reactions. Besides celiac disease and classical wheat allergy like baker’s asthma, a small proportion of wheat consuming subjects appears to suffer from non-celiac wheat sensitivity (NCWS; Schuppan et al. 2015; Catassi et al. 2017). NCWS can be defined as an adverse immune-mediated reaction to wheat products. Patients report intestinal or extra-intestinal symptoms, usually delayed by several hours after consumption of wheat. Wheat amylase trypsin inhibitors (ATIs) play a prominent role in the reactions. They are largely resistant to digestion by gastrointestinal proteases and activate intestinal innate immune cells, mainly macrophages and dendritic cells via the toll like receptor 4 (TLR4; Junker et al. 2012; Cuccioloni et al. 2017; Zevallos et al. 2017). In mouse models of disease, this causes an exacerbation of inflammatory bowel disease (Zevallos et al. 2017; Pickert et al. 2020), fatty liver disease (Ashfaq-Khan et al. 2019), classical food or respiratory allergies (Bellinghausen et al. 2019; Zevallos et al. 2019), and even Alzheimer’s disease (Dos Santos Guilherme et al. 2020). ATIs are major triggers of classical, immediate-type, immunoglobulin E (IgE)-inducing respiratory and food allergies (Salcedo et al. 2011; Schuppan et al. 2015; Kalunke et al. 2020), and importantly, are now implicated as allergens in a novel, highly prevalent type of IgE-negative food allergy, as a major cause of the irritable bowel syndrome that affects up to 15% of most societies (Fritscher-Ravens et al. 2014, 2019). This IgE-negative, likely eosinophil and T helper 2 cell-mediated intestinal wheat allergy represents a major fraction of patients with NCWS (Carroccio et al. 2013).

Wheat ATIs consist of three subfamilies: tetramers, dimers, and monomers (Silano et al. 1973, 1977; Oda et al. 1997; Altenbach et al. 2011) that are all included in the non-gluten (albumin) protein fraction. Over the past years, the major research emphasis regarding wheat protein families associated with digestive health has been on gluten (Caminero and Verdu 2019). This major wheat protein fraction is important for bread and pasta quality but contains also the epitopes for celiac disease with numerous studies investigating its distribution in different wheat species and cultivars (Call et al. 2020; Geisslitz et al. 2020; Pronin et al. 2020a). Roughly summarized, gluten content decreased slightly comparing old with modern wheat cultivars, and gluten composition changed toward more glutenins and less gliadins mainly due to the favorable processing properties of glutenins (Call et al. 2020; Pronin et al. 2020b). In contrast to gluten, ATIs have only recently come into the focus of clinical wheat research. Studies published prior to 2012 mainly related to characterization of the structure and the function of ATIs (Oda et al. 1997; Franco et al. 2002), and their important if not prominent role in immediate type, IgE-mediated respiratory wheat allergy (baker’s asthma). The discovery that ATIs activate immune cells via TLR4 in vitro and in vivo has greatly stimulated research related to their biochemistry, biology and physiological effects (Salcedo et al. 2011; Junker et al. 2012; Carroccio et al. 2013; Fritscher-Ravens et al. 2014, 2019; Fasano et al. 2015; Schuppan et al. 2015; Schuppan and Zevallos 2015; Catassi et al. 2017; Cuccioloni et al. 2017; Zevallos et al. 2017, 2019; Dinu et al. 2018; Reig-Otero et al. 2018; Tundo et al. 2018; Ashfaq-Khan et al. 2019; Bellinghausen et al. 2019; Schuppan and Gisbert-Schuppan 2019; Kalunke et al. 2020; Pickert et al. 2020). Moreover, ATI quantification in various wheats was performed using different extraction protocols (Prandi et al. 2013; Zevallos et al. 2017; Geisslitz et al. 2018; Bose et al. 2019). The currently largest study by Geisslitz et al. (2020) compared the content of 13 ATI proteins investigating eight cultivars among five wheat species, including hexaploid spelt (*T. aestivum ssp. spelta*), tetraploid durum (*T. turgidum ssp. durum*) and emmer (*T. turgidum ssp. dicoccum*), and diploid einkorn (*T. monococcum ssp. monococcum*) grown at three different environments, with very low concentrations of the measured ATI proteins in einkorn, while the other species containing a comparable level of total ATIs. However, significant variations for the different ATIs within each wheat species, with moderate heritabilities, were observed. Nevertheless, this study was based on only eight cultivars per species and estimates of genetic variances and heritabilities have normally large errors or confidence intervals. Moreover, to the best of our knowledge, environmental effects on ATI compositions have not yet been investigated in bread wheat, and studies on the genetic architecture of ATI proteins in wheat are lacking.

We therefore investigated a highly diverse set of 149 wheat cultivars tested at three different field locations for their amount of eight ATI proteins. Additionally, a genome-wide association mapping approach was performed using 22,220 Diversity Arrays Technology (DArT) markers. Our objectives were to (1) examine the genotype and the environment effect on the expression of eight major ATIs, (2) evaluate the correlation of these ATI proteins with important quality parameters, and (3) elaborate the genetic architecture underlying the expression of these eight ATIs.

## Materials and methods

### Plant material and field experiments

A total of 149 bread wheat (2*n* = 6*x *= 42, AABBDD) cultivars were used in this study, which were registered between 1921 and 2013 and originate from different European countries. The list of the cultivars and their details are provided in Table S1. Field trials were conducted in one winter cropping season (2015–2016) at three locations, Hohenheim (HOH, 48°43′07.3″N 9°11′08.7″E, altitude 403 m), Oberer Lindenhof (OLI, 48°28′19.0″N 9°18′29.3″E, altitude 700 m) and Eckartsweier (EWE, 48°32′52.4″N7°52′32.5″E, altitude 140 m) in Germany. Trials were arranged in partially replicated (P-rep) designs with a replication factor of 1.125 (Williams et al. 2011) and a net plot size of 1.25 m^2^. Plants were sown in October and harvested in July. During the growing season, standard cultivation practices were adopted of intensive wheat production applying 200 kg/ha of nitrogen fertilizer including Nmin, growth regulators and fungicides.

### Protein extraction

The seeds of all the wheat samples were cleaned using the Mini-Petkus seed cleaner and then milled using a laboratory mill equipped with a 1 mm sieve (Cyclotec 1093, FOSS, Hillerod, Denmark) to obtain the whole-grain flour. ATIs were quantitatively extracted from 1 g of whole-grain flour using 5 mL of extraction buffer (10 mM sodium bicarbonate, 500 mM sodium chloride, pH 7.8) with constant spinning at 4 °C overnight. The suspension was centrifuged at 4fSubsequently, the best linear unbiased 600 × *g* for 30 min, the supernatant collected and the procedure was repeated with an additional 5 mL of extraction buffer. Supernatants were combined and sterile-filtered (0.22 µm). Our prior study showed that this procedure extracted > 90% of ATIs, while maintaining their native protein conformation and solution stability (Sielaff et al. 2021).

### ATI quantification by liquid chromatography-mass spectrometry

Proteomic sample preparation, liquid chromatography-mass spectrometry (LC–MS) analysis, data processing and absolute quantification of ATIs using isotope-labeled standard peptides were performed as detailed before (Sielaff et al. 2021).

5 µL of flour extract was spiked with 30 pmol of ^13^C_6_-arginine- and ^13^C_6_-lysine-labeled ATI QconCAT protein, an artificial concatemer of standard peptides (Beynon et al. 2005) used for ATI quantification (Addgene plasmid # 163,957; http://n2t.net/addgene:163957; RRID: Addgene_163957). After denaturation using 0.1% (w/v) RapiGest (Waters Corporation, Milford, MA, USA), proteins were reduced, alkylated and digested overnight using MS grade trypsin (Promega, Madison, WI, USA) at a protease-to-protein ratio of 1:25 (w/w). RapiGest was hydrolyzed by addition of trifluoroacetic acid and precipitates were removed by centrifugation. Peptides were further desalted using Sep-Pak tC_18_ cartridges (Waters Corporation) and lyophilized. Prior to LC–MS analysis, peptides were dissolved in 20 µL of 0.1% (v/v) formic acid.

Furthermore, a reference mix of extracts was generated and used to titrate a dilution series from 40 to 0.625 µL. Each sample of the dilution series was spiked with 30 pmol of QconCAT protein and processed as described before. The reference samples were later used to generate external calibration curves for peptide quantification.

LC–MS analyses of tryptic peptides were performed using a nanoACQUITY UPLC system (Waters Corporation) connected to a SYNAPT G2-S mass spectrometer (Waters Corporation) via a NanoLockSpray dual electrospray ionization source (Waters Corporation). 0.5 µL of peptides were directly injected onto a HSS-T3 300 µm × 100 mm, 1.8 µm reversed-phase column (Waters Corporation) and eluted using a gradient mobile phase at a flow rate of 8 µL/min for 15 min. Mobile phase solvent A was water with 0.1% (v/v) formic acid. Solvent B was acetonitrile with 0.1% (v/v) formic acid, which was gradually increased from 1 to 36% (v/v) during elution. The column temperature was 55 °C. Post-column addition of 25% (v/v) dimethyl sulfoxide in acetonitrile at a flow rate of 1 µL/min was performed as described before (Distler et al. 2019). 250 fmol/µL [Glu1]-Fibrinopeptide B was directly infused into the electrospray ionization source via the reference sprayer using a flow rate of 1.5 µL/min.

Mass spectra were acquired by alternating between low (MS) and elevated energy scans (MS^E^). Acquisition time was 0.4 s in each scan with an interscan delay of 0.05 s. During MS scans, a constant collision energy of 4 eV was applied, while the collision energy was ramped from 16 to 40 eV during MS^E^ scans. The doubly charged monoisotopic ion of [Glu1]-Fibrinopeptide B was used as lock mass by sampling the reference sprayer in 30 s-intervals.

Raw data of standard samples were processed using ProteinLynx Global Server v3.0.2 (PLGS, Waters Corporation) and searched against a database containing *T. aestivum* proteins (UniProtKB release 2019_11, taxon ID: 4546, 142,969 entries + potential contaminants), specifying trypsin as protease, allowing two missed cleavages per peptide and defining carbamidomethylation as fixed and methionine oxidation as variable modification. In addition, isotope-labeled lysine and arginine were allowed as variable modification. The false discovery rate (FDR) was estimated by searching a reversed protein database and an FDR cut-off of 0.01 was applied.

The results were used to build a spectral library for the QconCAT peptides and unlabelled forms in Skyline v20.1.0.155. Afterward, targeted extraction of raw data, peak area integration, generation of external standard curves and absolute quantification at the precursor ion level were performed using Skyline as described in Sielaff et al. (2021).

The quantities of following ATI types were reported as average of indicated peptide quantities: monomeric ATI 0.28 (SVYQELGVR, LQCVGSQVPEAVLR), dimeric ATI 0.19 (DCCQQLAHISEWCR, LTAASITAVCR), dimeric ATI 0.19-like (VPALPGCRPVLK, LTAASITAVCK), tetrameric ATI CM1 (EYVAQQTCGISISGSAVSTEPGNTPR), ATI CM2 (EYVAQQTCGVGIVGSPVSTEPGNTPR), ATI CM3 (SGNVGESGLIDLPGCPR, YFIALPVPSQPVDPR), ATI CM16 (QQCCGELANIPQQCR, IETPGSPYLAK) and ATI CM17 (QECCEQLANIPQQCR, IEMPGPPYLAK). Units of measure are expressed as pmol per 5 µL of ATI extract used for proteomic analysis, equivalent to 0.5 mg of extracted wheat flour.

The mass spectrometry proteomics data have been deposited to the ProteomeXchange Consortium via the PRIDE (Perez-Riverol et al. 2019) partner repository with the dataset identifier PXD023654.

### Quality traits measurements

The analysis of the quality traits asparagine content (ASP), protein content (PC), sedimentation volume (SDS), sulfur content (SC), thousand kernel weight (TKW), test weight (TW) and falling number (FN) were described in detail in a previous study (Rapp et al. 2018).

### Statistical analysis

The phenotypic data were analyzed according to the following linear mixed model:

$$y_{{{\text{ijkl}}}} \; = \;\mu + g_{i} + {\text{env}}_{j} + g_{i} :\;{\text{env}}_{j} + r_{{{\text{jk}}}} + b_{{{\text{jkl}}}} + e_{{{\text{ijkl}}}}$$

where *y*_ijkl_ was the phenotypic observation of the *i*th genotype tested at the *j*th environment in the *k*th replication and in *l*th block, *µ* was the general mean, *g*_*i*_ was the genotypic effect of the *i*th genotype, env_*j*_ was the effect of the *j*th environment, *g*_*i*_ ∶ env_*j*_ was the genotype-by-environment interaction, *r*_jk_ was the effect of the *k*th replication at the *j*th environment, *b*_jkl_ was the effect of the *l*th block at the *k*th replication at the *j*th environment and *e*_ijkl_ was the residual error. Genotype, genotype by environment interaction and block effects were considered as random, and environment and replication as fixed effects model terms. The significance of random terms was tested by model comparison using a likelihood ratio test, and Wald test was used for significance of the fixed terms.

Subsequently, the best linear unbiased estimates (BLUEs) were estimated across the three environments (locations) assuming fixed genetic effects. The least significant difference (LSD) was determined for each trait at a significance level of 0.05. Broad sense heritability (*H*^2^) was calculated following Piepho and Möhring (2007) as: *H*^2^ = 1 − $~\frac{\vartheta }{{2\sigma _{G}^{2} }}$, where ϑ is the mean variance of a difference of two best linear unbiased predictors and $\sigma _{G}^{2} ~$ the genetic variance. Pearson correlation coefficients were estimated for all traits using the BLUEs across environments and the R package ‘corrplot’ (Wei and Simko 2017).

Box-and-whisker plots were constructed using the R package ggplot2 to investigate the distribution of the cultivar panel based on the year of registration and origin of the wheat cultivars. To test whether the ATI content has changed between the groups of registration periods and varietal origin, we compared the groups using non-parametric multiple comparison test Kruskall-Wallis with the R package ‘pgirmess’. All phenotypic analyses were conducted using the statistical software R (R Development Core Team 2016) and software package ASReml-R 3.0 (Gilmour et al. 2009).

### Genomic data and genotyping

Details of the genotyping approach are described in Rapp et al. (2018). Briefly, the cultivars were genotyped by genotyping-by-sequencing at Diversity Arrays Technology (Yarralumla, Australia) using the Wheat GBS 1.0 assay (DArTseq). DArTseq delivers two types of data, codominant SNP (S) and dominant DArT (D) markers. SNP and DArT markers showing more than 25% missing data and having minor allele frequencies lower than 5% were removed. The remaining missing markers were imputed using the package LinkImpute (Money et al. 2015). A total of 41,604 high-quality markers were obtained with only 22,122 markers having known genetic map positions (Li et al. 2015). Therefore, a chromosomal position was assigned to the significantly associated unmapped markers (98) based on the highest (*r*^2^ > 0.4) linkage disequilibrium (LD) with the mapped markers. Finally, we ended up with 22,220 markers with a map position. The physical positions of the markers were determined by BLASTing the marker sequences against the wheat genome IWGSC RefSeq v1.0 (The International Wheat Genome Sequencing Consortium 2018).

### Genome-wide association study (GWAS) and genomic prediction

GWAS was carried out using the R package GenABEL, (Aulchenko et al. 2007) where a mixed linear model was fitted incorporating marker data and a kinship matrix. Principal coordinates analysis did not show any clusters; therefore, principal coordinates were not included in the model. We used an explorative significance threshold of *P* < 0.001 and a Bonferroni-corrected threshold of *P* < 0.05 to identify significant marker-trait associations.

To obtain the genotypic variance explained by each quantitative trait locus (QTL) and across all QTL, we used a linear model fitting the significant QTL ordered based on the strength of their association. The explained genotypic variance (*p*_G_) was calculated as: *p*_G_ = $\frac{{R^{2} {\text{adj}}}}{{H^{2} }}$ (Utz et al. 2000; Würschum et al. 2016), where $R^{2} {\text{adj}}$ is the adjusted R^2^ from the linear model and H^2^ is the heritability of the trait. Only the most strongly associated markers, which explained more than 1% of the genotypic variance, were declared as putative QTL and reported in the manuscript.

Genomic prediction was conducted based on Ridge Regression best linear unbiased prediction (RR-BLUP) method with fivefold cross-validation using the R package ‘rrBLUP’ (Endelman 2011). In addition, weighted Ridge Regression-BLUP was performed by including the identified QTL explaining more than 10% of the genetic variance as fixed effects in the model (Zhao et al. 2014).

For candidate gene search, the latest publicly available wheat genome (IWGSC RefSeq v1.0) and gene functional annotation information were downloaded from the URGI database (Alaux et al. 2018; available at: https://wheat-urgi.versailles.inra.fr/Seq-Repository/Annotations). High (HC) and low confidence (LC) genes were extracted from the identified chromosomal regions of the significant QTL explaining more than 10% of genotypic variance. The genes with the functional annotation similar to the different domains of ATIs were selected as potential candidate genes.

## Results

### Phenotypic characterization of ATI proteins in wheat

**Fig. 1:**
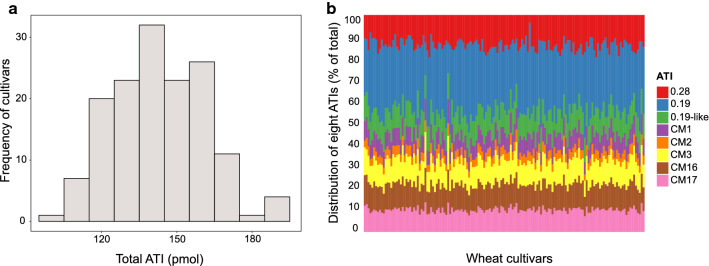
Variability of total ATI content, which is the sum of all eight measured individual ATI proteins **a** and the variability of ATI composition per cultivar by setting total ATI content to 100% **b** across the 149 investigated wheat cultivars

We detected a wide range of genotypic values for the content of all ATIs across the 149 wheat cultivars (Table 1). For instance, content of ATI 0.28 ranged from 4.96 pmol for cultivar 'Mv Zelma' to 31.77 pmol for cultivar 'Potenzial'. Similarly, a wide variation was determined for the content of ATI 0.19 ranging from 14.67 pmol for cultivar 'Expert' to 80.40 for cultivar 'Granada'. For the content of the other six investigated ATI proteins, differences were less pronounced, with a roughly twofold difference between minimum and maximum content. Summing up all eight ATI proteins, total ATI showed an up to twofold difference across the cultivars ranging from 98.55 pmol for cultivar 'Cezanne' up to 195.12 pmol for cultivar 'Slejpner' (Fig. 1a). The average contribution of single ATIs to average total ATI content across the 149 wheat cultivars was 31% ATI 0.19, 13% ATI 0.28, 12% ATI CM16, 11% ATI 0.19-like, 11% ATI CM3, 10% ATI CM17, 8% ATI CM1 and 4% ATI CM2. However, this percentage contribution of individual ATI proteins also largely varied across the 149 cultivars (Fig. 1b). For instance, (dimeric) ATI 0.19 contributed between 14 and 46% and tetrameric ATI CM16 contributed between 11.84 and 24.81% of the total ATI content among the 149 cultivars.

**Table 1:** Summary statistics for the eight ATI proteins (monomeric ATI 0.28, dimeric ATI 0.19 and ATI 0.19-like, tetrameric ATI CM1, ATI CM2, ATI CM3, ATI CM16, ATI CM17) measured among 149 bread wheat cultivars tested in three environments

Trait	ATI 0.28 (pmol)	ATI 0.19 (pmol)	ATI 0.19-like (pmol)	ATI CM1 (pmol)	ATI CM2 (pmol)	ATI CM 3 (pmol)	ATI CM 16 (pmol)	ATI CM 17 (pmol)	Total ATI (pmol)
Min	4.96	14.67	9.42	7.20	3.76	11.04	11.84	9.75	98.55
Mean	19.49	43.97	15.38	11.09	5.64	15.70	16.89	14.56	143.04
Max	31.77	80.40	21.96	16.82	9.64	22.12	24.81	22.44	195.12
LSD	5.67	26.36	4.47	2.64	1.46	4.70	5.12	3.92	43.40
$\sigma _{G}^{2}$	15.27***	49.27***	3.03***	1.04***	0.63***	2.10***	2.24***	2.21***	127.87**
$\sigma _{{G~ \times E}}^{2}$	0.09	0.00	0.00	0.58	0.27*	1.96	3.32*	1.39	87.30
$\sigma _{e}^{2}$	12.22	236.08	6.97	1.87	0.46	5.83	5.47	3.94	566.65
H^2^	0.79	0.37	0.55	0.55	0.70	0.44	0.42	0.54	0.36
E effect^†^	***	***	*	***	***	***	ns	**	***

LSD least significant difference at 5% probability level, $\sigma _{G}^{2}$ genotypic variance, $\sigma _{{G~ \times E}}^{2}$ genotype-by-environment interaction variance, $\sigma _{e}^{2}$ error variance, $H^{2}$ heritability

*, **, ***Significant at the 0.05, 0.01 and 0.001 probability levels, respectively

^†^Significance of the environment (E) effect according to Wald test, which was taken as fixed term in the statistical model; ns = non significant

These large differences led to highly significant genotypic variances for all eight ATIs and total ATI content (Table 1). For ATI 0.28, ATI 0.19 and ATI 0.19-like, very low variances due to genotype-by-environment interaction were determined. For the other ATI proteins and total ATI content, a considerable genotype-by-environment interaction was found. Furthermore, variances due to residual error were as high as genotypic variances or even higher for almost all ATIs. This led to low to medium estimates of broad sense heritability ranging from 0.36 for total ATI content to 0.79 for ATI 0.28 across all locations. At single locations, the heritabilities (repeatabilities) ranged from 0.14 for ATI 0.19-like in EWE to 0.81 for CM1 in OLI (data not shown). Except for all CM type ATIs, correlation coefficients across different ATI proteins were low (Table 2). Similarly, all ATIs showed only weak correlations with important quality traits commonly assessed in wheat breeding (Table 2).

**Table 2:** Pearson’s correlation coefficients among eight measured ATI proteins as well as between ATI proteins, total ATI content and quality traits assessed using the same set of bread wheat cultivars tested in three environments

	ATI 0.28	ATI 0.19	ATI 0.19-like	ATI CM1	ATI CM2	ATI CM3	ATI CM16	ATI CM17	Total ATI
ATI 0.28									
ATI 0.19	0.16								
ATI 0.19-like	0.28***	0.42***							
ATI CM1	0.18*	0.27***	0.41***						
ATI CM2	0.10	0.03	0.28***	0.85***					
ATI CM3	0.28***	0.15	0.3***	0.83***	0.78***				
ATI CM16	0.21**	0.37***	0.35***	0.78***	0.69***	0.83***			
ATI CM17	0.18*	0.14	0.31***	0.87***	0.87***	0.87***	0.77***		
Total ATI	0.47***	0.81***	0.62***	0.68***	0.47***	0.62***	0.72***	0.60***	
ASP	− 0.05	0.09	− 0.19*	− 0.14	− 0.07	− 0.16	− 0.09	− 0.10	− 0.03
PC	0.10	0.19*	0.04	0.17*	0.20*	0.11	0.14	0.20	0.23*
SDS	− 0.02	0.05	− 0.03	− 0.06	− 0.12	− 0.08	− 0.02	− 0.12	− 0.02
SC	0.10	0.18*	− 0.05	0.39***	0.40***	0.41***	0.39***	0.39***	0.32***
TKW	− 0.09	− 0.21**	− 0.09	0.01	0.09	0.19*	0.07	0.07	− 0.12
TW	0.04	0.01	− 0.14	− 0.01	− 0.11	0.05	0.12	− 0.03	0.01
FN	0.09	− 0.07	− 0.03	− 0.19*	− 0.09	− 0.20	− 0.13	− 0.11	− 0.10

ASP asparagine content; PC protein content; SDS sedimentation volume; SC sulfur content; TKW thousand kernel weight; TW test weight; FN falling number

*, **, ***Significant at the 0.05, 0.01 and 0.001 probability levels, respectively

**Fig. 2:**
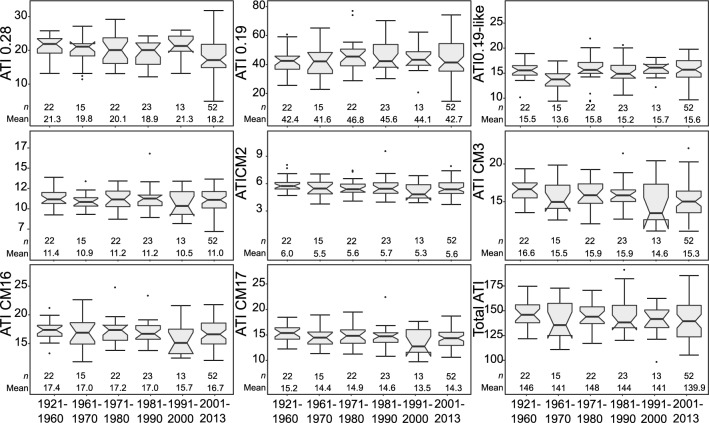
Boxplots showing the amount of different ATI proteins in the wheat cultivars depending on the period, when the respective wheat cultivars were officially registered. Numbers in the plot indicate the mean values and the number of cultivars in each group. Two cultivars with lacking registration information were omitted

The investigated wheat cultivars were registered from 1921 to 2013 and originate from different European countries. We split them in different groups for time periods between 1921 and 1960 and from 1961 onward in decades leading to group sizes between 13 and 23 except for the last decade, where 52 varieties were assessed (Fig. 2). We found a large variability for individual ATIs and for total ATI content in each time period, and no temporal trend across the decades. Similarly, we formed groups of origin across seven regions in Europe (Fig. S1), but group sizes largely varied with an imbalance due to a large number of German cultivars. In each group, we also found a wide variation in ATI contents but no regional trend. According to the Kruskall-Wallis test, no significant difference (for *p* < 0.05) between the grouping either for registration years or origin was found.

### The genetic architecture underlying ATI protein content

**Fig. 3:**
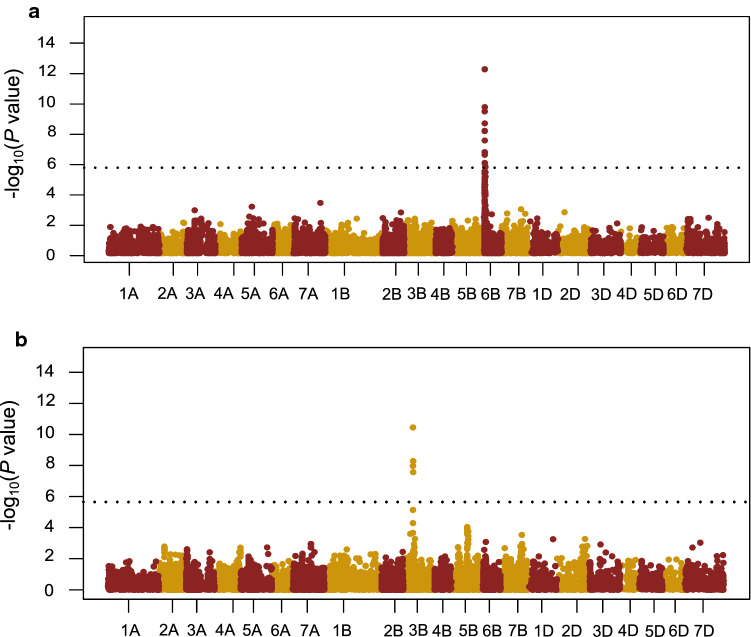
Manhattan plots showing significant marker-trait associations for ATI 0.28 (**a**) and ATI 0.19-like (**b**) at Bonferroni-corrected significance threshold of *P* < 0.05. The x-axis shows the DArTseq markers on 21 chromosomes based on the genetic map positions (cM) and the y-axis shows the *P* values on a − log_10_ scale

A genome-wide association mapping using 22,220 polymorphic markers delivered a total of 68 QTL significant at *p* < 0.001 across the eight ATIs and total ATI content (Table S2, Fig. S2). Among them, 30 QTL explained more than 10% of the genotypic variation for ATI 0.28 on chromosome 6B, for ATI 0.19 on chromosomes 1B and 3B, for ATI 0.19-like on chromosome 3B, for ATI CM1 on chromosomes 1A, 1B and 4B, for ATI CM2 on chromosomes 1B, 7B and 1D, for ATI CM3 on chromosomes 1A, 5A, 4B and 6D, for ATI CM16 on chromosomes 5A, 6B and 7B, for ATI CM17 on chromosomes 1B, 4B and 7D as well as for total ATI on chromosomes 1A, 6A, 1B, 2B, 3B and 2D. Furthermore, QTL in two chromosomal regions surpassed the Bonferroni significance threshold of *p* < 0.05 (–log_10_ *p* value = 5.65; Table 3, Fig. 3). These two major QTL explained 68 and 71% of the genotypic variation for ATI 0.28 and ATI 0.19-like, respectively.

**Table 3:** Marker-trait associations surpassing Bonferroni threshold (5.65) indicating putative quantitative trait loci (QTL) for ATI 0.28 and ATI 0.19-like

Trait	Marker	Chr	Gen Pos (cM)	Phy Pos (bp)	LOD	*P*_G_	α-Effect
ATI 0.28	1106155D	6B	6.45	2,094,331	13.71	68.73	3.30
ATI 0.19-like	2275974D	3B	93.78	78,749,798	10.45	70.55	− 2.67
	1123753S	3B	93.78	78,136,767	7.97	7.58	− 1.91

Chr. Chromosome, Gen Pos chromosome position in cM, Phy Pos sequence start position in base pairs according to the bread wheat reference genome (IWGSC RefSeq v1.0), *P*_G_ proportion of genotypic variance explained by the QTL in percent, and allele substitution (α) effect

**Fig. 4:**
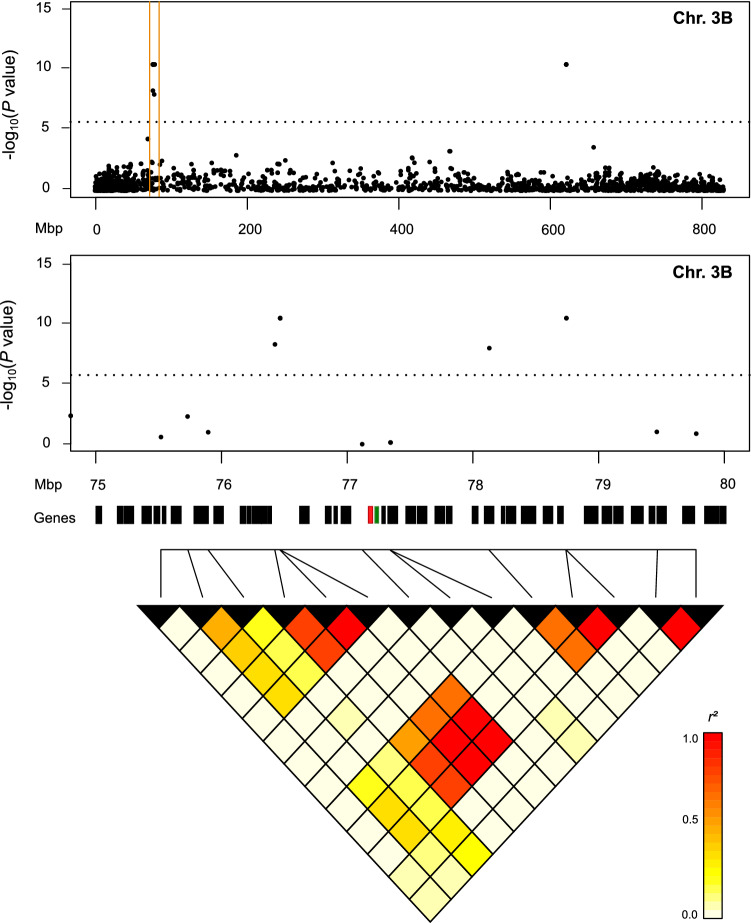
Fine-mapping of the major QTL (2275974D) for ATI 0.19-like on chromosome 3B. Genes detected in the target region 75–80 Mbp on chromosome 3B are marked by black boxes, while the two potential candidate genes TraesCS3B02G111100 and TraesCS3B02G111200 are marked by red and green boxes, respectively. Linkage disequilibrium (*r*^*2*^) among all the markers in the region 75–80 Mbp is shown in the heatmap

After this initial genome scan, we investigated in more detail the chromosomal regions harboring the 30 significant QTL, which explained more than 10% of the genotypic variation (Fig. 4, Fig. S3). For these regions, we extracted high (HC) and low confidence (LC) genes from the bread wheat reference genome (IWGSC RefSeq v1.0) and evaluated these as potential candidate genes with functional annotations similar to the different domains of ATIs. With that approach, eight potential candidate genes were identified out of a total of 1,081 genes harbored in the target regions (Table 4, Table S3). These candidate genes were TraesCS3B02G111100 and TraesCS3B02G111200 for ATI 0.19-like located between 75 and 80 Mbp on chromosome 3B, TraesCS1A02G048700 for ATI CM1 located in the region 29.5–30.5 Mbp of chromosome 1A, TraesCS7B02G072000 located in the region between 72 and 85 Mbp on chromosome 7B, TraesCS1D02G163900 and TraesCS1D02G164000 located on chromosome 1D between 234 and 235.5 Mbp for ATI CM2, as well as TraesCS3B02G170800 located on chromosome 3B between 171 and 174 Mbp for total ATI content. No potential candidate genes with annotations in the Pfam and InterPro databases with relation to ATI could be identified for the QTL detected for the ATI proteins 0.28, 0.19, CM3, CM16 and CM17. Finally, we investigated the linkage disequilibrium (LD) pattern in the chromosomal region of the two major QTL for ATI 0.28 and ATI 0.19-like (Fig. 4, Fig. S3). The region of the major QTL detected for ATI 0.19-like on chromosome 3B harbors many genes (63) including the two candidate genes TraesCS3B02G111100 and TraesCS3B02G111200 (Fig. 4). In contrast to our expectation of a LD pattern with high LD between our significant markers and the markers being closest to the two candidate genes in that small chromosomal region, we determined a break of LD pattern within this region. The markers closest to the candidate genes were only in very weak LD to the two highly significant markers. For the major QTL on chromosome 6B for ATI 0.28, we could neither find any potential candidate genes with an annotation similar to an ATI nor a strong LD pattern in that chromosomal region (Fig. S3).

**Table 4:** List of eight candidate genes identified for ATI 0.19-like, ATI CM1, ATI CM2 and total ATI content through functional analysis on all mapped QTL explaining more than 10% of genotypic variance

Trait	GeneID	Pfam^a^	InterPro^a^	Chr	start	end
*ATI 0.19-like*						
	TraesCS3B02G111100	PF00234: Protease inhibitor/seed storage/LTP family	**IPR006105**: Cereal seed allergen/trypsin and alpha-amylase inhibitor, conserved site;	chr3B	77,315,338	77,316,027
			**IPR006106**: Cereal seed allergen/grain softness/trypsin and alpha-amylase inhibitor;			
			**IPR016140**: Bifunctional inhibitor/plant lipid transfer protein/seed storage helical domain;			
			**IPR016309**: Alpha-amylase inhibitor/seed allergen			
	TraesCS3B02G111200	PF00234: Protease inhibitor/seed storage/LTP family	**IPR006105**: Cereal seed allergen/trypsin and alpha-amylase inhibitor, conserved site;	chr3B	77,349,935	77,350,623
			**IPR006106**: Cereal seed allergen/grain softness/trypsin and alpha-amylase inhibitor;			
			**IPR016140**: Bifunctional inhibitor/plant lipid transfer protein/seed storage helical domain;			
			**IPR016309**: Alpha-amylase inhibitor/seed allergen;			
	TraesCS3B02G294800	PF14368: Probable lipid transfer	**IPR016140:** Bifunctional inhibitor/plant lipid transfer protein/seed storage helical domain	chr3B	4.74E + 08	4.74E + 08
*ATI CM1*						
	TraesCS1A02G048700	PF14368: Probable lipid transfer	**IPR000528**: Plant lipid transfer protein/Par allergen;	chr1A	30,196,596	30,197,675
			**IPR016140**: Bifunctional inhibitor/plant lipid transfer protein/seed storage helical domain			
*ATI CM2*						
	TraesCS7B02G072000	PF00234: Protease inhibitor/seed storage/LTP family	**IPR006105**: Cereal seed allergen/trypsin and alpha-amylase inhibitor, conserved site;	chr7B	79,308,131	79,309,947
			**IPR006106**: Cereal seed allergen/grain softness/trypsin and alpha-amylase inhibitor;			
			**IPR016140**: Bifunctional inhibitor/plant lipid transfer protein/seed storage helical domain;			
	TraesCS1D02G163900	PF14368: Probable lipid transfer	**IPR016140**: Bifunctional inhibitor/plant lipid transfer protein/seed storage helical domain;	chr1D	2.35E + 08	2.35E + 08
	TraesCS1D02G164000	PF14368: Probable lipid transfer	**IPR016140**: Bifunctional inhibitor/plant lipid transfer protein/seed storage helical domain;	chr1D	2.35E + 08	2.35E + 08
**Total ATI**	TraesCS3B02G170800	PF00234: Protease inhibitor/seed storage/LTP family	**IPR000528**: Plant lipid transfer protein/Par allergen;	Chr3B	1.72E + 08	1.72E + 08
			**IPR016140**: Bifunctional inhibitor/plant lipid transfer protein/seed storage helical domain			

^a^ Protein families database

Finally, we performed a genome-wide prediction approach for all eight ATIs and total ATI content (Fig. S4). For all ATIs, the cross-validated prediction ability was already high using marker-assisted selection based only on the QTL explaining more than 10% of the genotypic variance. Combining these markers with RR-BLUP slightly improved the cross-validated prediction abilities. The highest average prediction ability was achieved with *r* = 0.76 for ATI 0.28 and the lowest with *r *= 0.43 for ATI 0.19.

## Discussion

### Wheat cultivars largely differ in their content and composition of ATIs

The investigated 149 wheat cultivars largely differed in their contents of the eight evaluated (major) ATIs as well as their total ATI content (Table 1, Fig. 1a). For instance, the content of ATI 0.28 and ATI 0.19 varied more than fivefold between the different cultivars, while for the other ATIs and total ATI content, this variation was up to two- or threefold. Furthermore, contents of the different ATIs were only partly correlated across the cultivars. For all measured tetrameric CM type ATIs, high coefficients of correlation were found (Table 2). Thus, a wheat cultivar low in one CM type ATI trended to be also low in the other CM type ATIs. By contrast, coefficients of correlation between ATI 0.19, ATI 0.19-like and ATI 0.28 as well as between them all and all CM type ATIs were quite low. For instance, the lowest content of ATI 0.28 was identified for the Hungarian cultivar 'Mv Zelma' but with a relatively high content of ATI 0.19. These differences led to a large variation in ATI composition in the different wheat cultivars (Fig. 1b). While few cultivars were quite low across most ATIs leading also to a low total ATI content (e.g., 'Cezanne', 'Akteur', 'Hermann'), others were low in one ATI but high in other ATIs (e.g., 'Mv Zelma', 'Skater'). These results are in agreement with findings of Bose et al. (2020), who compared 23 hexaploid wheat lines for 18 ATIs, Geisslitz et al. (2020) and Call et al. (2020), who quantified different ATIs in few cultivars across different wheat species. These authors have shown a wide variability in ATI content and composition across the investigated cultivars. Consequently, there is the potential to change contents and composition of ATIs in wheat by selecting specific cultivars.

The success of this choice along the wheat supply chain, however, depends besides other factors on the extent to how much the expression of the single ATI proteins is influenced by the environmental conditions, where the cultivars are grown. In particular, only traits with high heritability (at least 0.6, and larger) were stably expressed across different environments and growing conditions and can therefore be manipulated across supply chains via choice of cultivars. For six out of eight ATIs, we determined medium to low heritability values (Table 1), showing the strong environmental impact. By contrast, for ATI 0.28 and ATI CM2, heritabilities of 0.70 and 0.79 were obtained, respectively. For commonly investigated quality traits that are important for milling and bread making quality, heritability values ranged from 0.65 for protein content up to almost 0.9 for sedimentation volume, hectoliter weight and thousand kernel weight measured on the same samples (Rapp et al. 2018). This underlines clearly that most of ATI proteins seem to be strongly influenced by environmental conditions as was also demonstrated by Prandi et al. (2013) and are therefore difficult to be manipulated along global wheat supply chains via choice of cultivar. Interestingly, the high heritability values obtained for ATI 0.28 and ATI CM2 confirm results from Geisslitz et al. (2020). By contrast, our heritability estimates for the other ATIs, especially 0.19, CM1 and CM3, are lower than the values reported by Geisslitz et al. (2020). As estimates of variance components and heritabilities have large errors, and as Geisslitz et al. (2020) used only eight varieties for bread wheat, we speculate that their heritability values might have been overestimated. Thus, the amount of ATI 0.28 and ATI CM2 might be influenced quite successfully along supply chains by choosing cultivars with low ATI 0.28 and CM2 content. However, total ATI content will most likely not change much, since these two ATIs contributed only 4–13% to the total ATI content. Furthermore, Call et al. (2020) have only shown an intermediate correlation coefficient of *r* = 0.61 between ATI content and their trypsin inhibitory activity. Hence, more research is needed on biological relevant activities and allergenicity of different ATI proteins as well as possibilities to manipulate these activities along the wheat supply chain through agronomic and other technological processes. Very recent studies investigated the contribution of food processing in ATI degradation by using different sourdough fermentations with lactic acid bacteria (Fraberger et al. 2020), or by comparing different bread making processes (Huang et al. 2020). Both studies showed the ability of sourdough fermentation to degrade ATI proteins for more than 22%. Moreover, Huang et al. showed a significant decrease in pro-inflammatory bioactivity of ATI underlining the potential of the bread making process on ATI.

### Breeding wheat cultivars for lower ATI content

Selecting between registered wheat cultivars those with appropriate ATI profiles might be seen as a potential strategy to reduce allergenic and pro-inflammatory ATI content. Thus, targeted wheat breeding might contribute to reduce NCWS. Prerequisites for a successful breeding for low ATI content are an existing genetic variance within the elite wheat germplasm, high heritabilities and no negative correlations with important agronomic and quality traits. As already discussed above, we determined a large genetic variance for all the measured eight ATI proteins but higher heritabilities only for ATI 0.28 and ATI CM2. Notably, correlation coefficients with important quality traits in wheat were for all ATIs close to zero (Table 2), indicating that breeding for lower ATIs would not hinder selection for baking quality. Thus, wheat breeding could contribute to a reduction in ATI content, but selection gain is limited for most ATI proteins except ATI 0.28 and ATI CM2, due to low heritability for the other ATIs. Furthermore, the recent methodology to determine ATI content is work and cost intensive as well as too slow for a high sample throughput, which is needed in intermediate stages of large wheat breeding programs. Thus, the development of a fast method, such as a simple enzyme linked immune assay (ELISA), would be crucial warranting further research.

Interestingly, we determined also low correlation coefficients between ATI contents and protein content determined by NIRS (ICC standard method 159, ICC, Vienna, Austria), which confirms another recent study (Geisslitz et al. 2020). Consequently, a high protein content does not automatically imply a high ATI content that for now needs to be measured independently for all existing ATI proteins. Surprisingly, correlation coefficients between ATI proteins and falling number were close to zero (Table 2). The falling number is widely applied in wheat supply chains as an indirect method to determine the α-amylase activity. Similarly, Call et al. (2020) have shown only an intermediate correlation coefficient between ATI content and trypsin inhibitory activity. Consequently, more research is needed on the role and effects of ATI in cereals and cereal products.

The 149 investigated wheat cultivars of our study were registered from 1921 to 2013 and originated from different European countries. We therefore investigated the possible existence of temporal or regional trends in the ATI content of different wheat cultivars. For all eight ATI proteins as well as total ATI content, there was neither a temporal (Fig. 2) nor a regional trend (Fig. S1), showing that wheat breeders did not select for changes in ATI contents in Central and Eastern Europe in the last century, neither directly nor indirectly via correlation with other traits. This confirms our experience from many discussions with breeder colleagues and is also in accord with a study that found no significant difference between old and modern Austrian wheat evaluated for total ATI content (Shewry et al. 2020). This is in contrast to statements that modern bread wheat cultivars have increased ATI content compared to old varieties as originally hypothesized by Junker et al. (2012), and now widely stated in popular press and social media.

Furthermore, the absence of a trend over time and geographic regions regarding ATI content is also remarkable, since it is often claimed that breeders have actively enhanced ATIs in wheat due to insect resistance breeding. Beside the lacking trend of increased ATI content in modern wheat cultivars, wheat breeders have yet not invested much in insect resistance breeding except for orange blossom midge resistance (McKenzie et al. 2002), which has received increased attention within the last years. Amylase (trypsin) inhibitors naturally exist in wheat and might inhibit proteinases from insects such as weevils. Several early studies (Buonocore et al. 1980; Ryan 1990; Carbonero et al. 1999) reported amylase inhibitors to be active against storage pests such as *Tenebrio molitor.* However, to the best of our knowledge, the functions of ATIs in wheat are not yet sufficiently explored and warrant further research. Summarizing, to date, wheat breeding has not altered ATI contents and composition of wheat cultivars.

### ATI proteins are influenced by many small and few major QTL

The genome-wide association mapping identified between four (ATI CM1, ATI CM17) to 13 QTL (ATI CM16) for the eight investigated ATIs (Table 3, Table S2). Many of these QTL explained only a small proportion of the genotypic variance, which is common for quantitatively inherited traits. However, for each ATI at least one major QTL explaining > 23% of the genotypic variance was identified with two very large-effect QTL on chromosomes 6B and 3B explaining 68.7% and 70.6% of the genotypic variance for ATI 0.28 and ATI 0.19-like, respectively. We therefore tried to identify candidate genes with functional annotations similar to the different domains of ATIs based on the reference sequence (IWGSC RefSeq v1.0). We searched for all 30 QTL which explained > 10% of the genotypic variance, but could only determine eight potential candidate genes for QTL of ATI 0.19-like, ATI CM1, ATI CM2, and for total ATI content (Table 4). Notably, all these loci encoded wheat proteins of the lipid transfer protein family that are structurally related to ATI proteins (Asero et al. 2001). For the very large-effect QTL explaining 68.7% of the genotypic variance of ATI 0.28, we could not identify any gene in that chromosomal region with functional annotations similar to an ATI (Fig. S3). The chromosomal region of the major QTL detected for ATI 0.19-like harbors two potential candidate genes TraesCS3B02G111100 and TraesCS3B02G111200 (Fig. 4). However, a LD breakage within the peak region prevents clear conclusions. Thus, further research is needed to clarify the genetic architecture of ATI proteins in more detail across wider germplasm or within biparental mapping families.

The same chromosomal groups 3 and 6 were reported to mainly influence dimeric (0.19) and monomeric (0.28) ATIs, respectively. Earlier studies have reported that dimeric ATIs including ATI 0.19 were mapped to 3B (Sanchez-Monge et al. 1986; Singh and Skerritt 2001; Juhász et al. 2018). Similarly, ATI 0.28 was reported to be encoded by a single chromosomal region on 6D (Sanchez-Monge et al. 1986; Singh and Skerritt 2001; Bose et al. 2020). However, the mapping approach was different compared to our study and no information related to exact chromosomal location has been reported. Therefore, it is quite difficult to align the results from those studies to the current one. In contrast to the prior results, we additionally found further QTL for both ATIs on different chromosomes (Table S2). Furthermore, for the remaining six ATI proteins and total ATI content, we identified only medium to small effect QTL in different chromosomal locations in addition to the previously reported chromosomes group 4 and 7 for CM type ATIs (Fra-Mon et al. 1984; Salcedo et al. 1984). Consequently, the genetic architecture of ATI proteins appears more complex than reported and requires further research.

We identified high positive correlation coefficients for the expression of CM type ATIs (Table 2). Consequently, several CM type ATIs were controlled by co-localized QTL. A recent proteome study confirmed the high similarity of some ATIs including CM16 and CM17 by detecting common peptides after trypsin digestion (Geisslitz et al. 2020). Cluster analysis of ATI-like proteins by mean of discovery proteomics on wheat extracted proteins revealed that CM proteins fall into the same cluster (Bose et al. 2020). This makes physiological sense, since CM ATIs form stable noncovalently tetramers with two molecules of CM3 associating with two other CM ATIs (Gomez et al. 1989; Altenbach et al. 2011). Thus, the association within CM type ATI proteins seems systematic and further research is needed to elucidate this association in more detail. For instance, whether this association results from linked or pleiotropic genes, or similar pathways of gene or post-translational regulation.

Overall, the findings on the genetic architecture of ATI proteins can facilitate targeted wheat breeding. First, single marker assays could be developed for the major QTL on chromosome 3B and 6B for ATI 0.19-like and ATI 0.28, respectively. As both alleles at these QTL are widely present in modern cultivars (data not shown), this would allow an easy fixation of the favorable alleles across early generation selection. Secondly, we determined medium to high prediction abilities for all ATI proteins and total ATI content from genomic selection using either marker-assisted selection based only on QTL explaining > 10% of the genotypic variance or weighted RR-BLUP (Fig. S4). These prediction abilities are comparable with those of other traits in wheat, such as asparagine content, protein content and grain yield (Rapp et al. 2018; Michel et al. 2019) and high enough to increase annual selection gain by use of genomic selection (Marulanda et al. 2016). An efficient application in wheat breeding would require further research extending genomic calibrations on a wider range of cultivars coming from different breeding programs. However, starting to implement low ATI content as new trait in wheat breeding is an expensive and long-term decision with limited success due to the low heritability of many ATI proteins. Thus, we would only recommend it if medical and market necessities are further confirmed and if other alternatives such as milling and bread making technologies turn out to be less efficient than breeding in reducing ATI content along wheat supply chains. On the other hand, gene editing technologies might be another alternative to decrease content and unwanted biological activities of ATIs (Camerlengo et al. 2020; Kalunke et al. 2020). However, this is applicable only if target ATIs are encoded by major loci which appear not to be the case for most of them according to the current study.

## Conclusions

Based on the evaluation of eight major ATI proteins in a diverse collection of wheat cultivars, we could clearly show that wheat breeding has not altered the content and composition of ATI proteins neither directly nor indirectly in the last century of breeding history in Central Europe. We identified large differences in the content and composition of the ATI proteins in the different cultivars, and heritability values were high only for ATI 0.28 and ATI CM2. Thus, these two proteins bear the potential to be successfully manipulated via varietal choice across international wheat supply chains, but they contributed only up to 13% of total ATI content in our study. The genetic architecture of ATI proteins appears complex with many minor but few major QTL with large effect. Based on the wheat reference sequence, we could only identify eight potential candidate genes behind all major QTL that warrant future research. Additionally, further research is needed to validate the impact of ATI proteins that are major allergens, also on other aspects of human health. Furthermore, rapid methods must be elaborated to quantify either ATI proteins or determine ATI pro-inflammatory bioactivity in cereals or food samples within a short time. Eventually, the possibility to reduce ATI content and activity in wheat products must then be extended along the wheat supply chain to milling and bread or pasta making technology, including different dough fermentations or the use of enzymes to inactivate ATIs in wheat products in order to elaborate more efficient methods than selecting cultivars for low heritable traits.

## Supplementary Information

Below is the link to the electronic supplementary material.

Supplementary file1 (DOCX 5618 KB)

Supplementary file2 (EPS 153 KB)

Supplementary file3 (EPS 71498 KB)

Supplementary file4 (EPS 985 KB)

Supplementary file5 (EPS 126 KB)

Supplementary file6 (XLSX 152 KB)
