# Supplementary material for: Genetic architecture underlying the expression of eight α-amylase trypsin inhibitors
Source: Theor Appl Genet. 2021 Jul 10;134(10):3427–41. doi: 10.1007/s00122-021-03906-y (PMC8440294; doi:10.1007/s00122-021-03906-y)
Supplement: Supplementary file 1 — Supplementary file1 (DOCX 5618 KB) [file 122_2021_3906_MOESM1_ESM.docx]

**Genetic architecture underlying the expression of eight α-amylase trypsin inhibitors ~~as potential triggers of baker’s asthma and non-celiac wheat sensitivity~~**

Theoretical and Applied Genetics

Khaoula El Hassouni^1^, Malte Sielaff^2^, Valentina Curella^3^, Manjusha Neerukonda^3^, Willmar Leiser^1^, Tobias Würschum^4^, Detlef Schuppan^3,5,a^, Stefan Tenzer^2,a^, C. Friedrich H. Longin^1,a,*^

^1^ State Plant Breeding Institute, University of Hohenheim, Fruwirthstr. 21, 70599, Stuttgart, Germany

^2^ Institute for Immunology, University Medical Center of the Johannes Gutenberg-University Mainz, Langenbeckstr. 1, 55131 Mainz, Germany

^3^ Institute of Translational Immunology and Research Center for Immune Therapy, University Medical Center of the Johannes Gutenberg-University Mainz, Langenbeckstr. 1, 55131 Mainz, Germany

^4^Institute of Plant Breeding, Seed Science and Population Genetics, University of Hohenheim, Fruwirthstr. 21, 70599 Stuttgart, Germany

^5^Division of Gastroenterology, Beth Israel Deaconess Medical Center, Harvard Medical School, 330 Brookline Ave, Boston, MA 02215, USA

^a^ ST and DS share senior authorship with FL

^*^ Corresponding author: [friedrich.longin@uni-hohenheim.de](mailto:friedrich.longin@uni-hohenheim.de)

**Table S1** List of 149 bread wheat cultivars used in this study, their origin and year of registration of the cultivar

| Cultivar | Origin | Year of registration |
| --- | --- | --- |
| Admiral | Germany | 1968 |
| Akteur | Germany | 2003 |
| Alba | Poland | 1987 |
| Albatros | Belgium | 1974 |
| Alchemy | Great Britain | 2006 |
| Alidos | Germany | 1987 |
| Altigo | France | 2007 |
| Apache | France | 1999 |
| Aquila | Germany | 1981 |
| Ares | Germany | 1983 |
| Arezzo | France | 2007 |
| Aristos | Germany | 1997 |
| Aron | Germany | 1993 |
| Ataro | Switzerland | 2004 |
| Avalon | Great Britain | 1980 |
| Basalt | Germany | 1980 |
| Beaver | Great Britain | 1989 |
| Borenos | Germany | 1987 |
| Brilliant | Germany | 2005 |
| Bussard | Germany | 1990 |
| Butaro | Germany | 2009 |
| Cadenza | Great Britain | 1992 |
| Calif | Germany | 1983 |
| Campari | Germany | 2003 |
| Carenius | Germany | 2006 |
| Carimulti | Germany | 1975 |
| Carisuper | Germany | 1975 |
| Carstens VIII | Germany | 1952 |
| Cezanne | France | 1997 |
| Chevalier | Germany | 2005 |
| Claire | Great Britain | 1999 |
| Colonia | Germany | 2011 |
| Compliment | Germany | 2001 |
| Cubus | Germany | 2002 |
| Dekan | Germany | 1999 |
| Diplomat | Germany | 1966 |
| Discus | Germany | 2007 |
| Drifter | Germany | 1999 |
| Elite Lepeuple | France | 1954 |
| Erla Kolben | Austria | 1961 |
| Escorial | Netherland | 1987 |
| Etoile de Choisy | France | 1950 |
| Euclide | France | 2007 |
| Event | Germany | 2009 |
| Expert | France | 2007 |
| Extrem | Austria | 1967 |
| Fanal | Germany | 1953 |
| Flame | Great Britain | 1995 |
| Flamingo | Germany | 1960 |
| Florian | Germany | 1960 |
| Florida | Germany | 1985 |
| Galahad | Great Britain | 1983 |
| Genius | Germany | 2010 |
| Glatter Breisgauer Landweizen | Germany | - |
| Glaucus | Germany | 2011 |
| Granada | Germany | 1980 |
| Heines IV | Germany | 1940 |
| Heines VII | Germany | 1950 |
| Hermann | Germany | 2004 |
| Hobbit | Great Britain | 1977 |
| Holdfast | Great Britain | 1936 |
| Inspiration | Germany | 2007 |
| JB Asano | Germany | 2008 |
| JossCambier | France | 1966 |
| Jularo | Germany | 2008 |
| Julius | Germany | 2008 |
| Kleinwanzlebener | Germany | 1943 |
| Knirps | Germany | 1985 |
| Kobold | Germany | 1978 |
| Konsul | Sweden | 1990 |
| Kormoran | Germany | 1960 |
| Kraka | Denmark | 1981 |
| Kredo | Germany | 2009 |
| Lear | Great Britain | 2007 |
| Leda | Belgium | 1954 |
| Magister | Germany | 2006 |
| Marco | Italy | 1969 |
| Maris Huntsman | Great Britain | 1971 |
| Matrix | Germany | 2010 |
| Mega | Great Britain | 1972 |
| Minister | Belgium | 1947 |
| Miras | Germany | 1984 |
| Mironowskaja Jubilejnaja | ex-Yugoslavia | 1972 |
| Monopol | Germany | 1975 |
| Muck | Germany | 1962 |
| Mulan | Germany | 2006 |
| Multiweiss | Austria | 1965 |
| Mv Lucia | Hungary | 2007 |
| Mv Zelma | Hungary | 2007 |
| Naturastar | Germany | 2002 |
| Nautica | Netherland | 1977 |
| Nimbus | Germany | 1975 |
| Obelisk | Netherland | 1985 |
| Oberst | Germany | 1980 |
| Okapi | Germany | 1978 |
| Orcas | Germany | 2010 |
| Orlando | Germany | 1970 |
| Palur | Germany | 1986 |
| Pamier | Germany | 2008 |
| Pantus | Germany | 1966 |
| Paragon | Great Britain | 1998 |
| Patras | Germany | 2012 |
| Perlo | Austria | 1978 |
| Pionier | Germany | 2013 |
| Potenzial | Germany | 2006 |
| Progress | Germany | 1969 |
| Rabe | Germany | 1962 |
| Rektor | Austria | 1984 |
| Renan | France | 1989 |
| Riband | Great Britain | 1987 |
| Rimpaus Braun | Germany | 1939 |
| Santiago | Great Britain | 2011 |
| Saturn | Germany | 1973 |
| Schamane | Germany | 2005 |
| Schweigers Taca | Germany | 1942 |
| Severin | Germany | 1980 |
| Skalmeje | Germany | 2003 |
| Skater | Germany | 2000 |
| Slejpner | Sweden | 1986 |
| Solehio | Italy | 2008 |
| Solstice | Great Britain | 2002 |
| Sorbas | Germany | 1985 |
| Stamm 101 | Austria | 1951 |
| Starke II | Sweden | 1968 |
| Steadfast | Great Britain | 2002 |
| Svale | Sweden | 1956 |
| Svaloefs Bora | Sweden | 1947 |
| Tabasco | Germany | 2006 |
| Tabor | Germany | 1979 |
| Tadorna | Netherland | 1966 |
| Taras | Germany | 1982 |
| Tengri | Switzerland | 2007 |
| Terrier | Germany | 2001 |
| Tobak | Germany | 2011 |
| Tommi | Germany | 2002 |
| Topfit | Germany | 1972 |
| Toras | Germany | 2004 |
| Transit | Germany | 1994 |
| Tremie | France | 1992 |
| Tschermaks Weisser Begrannter | Austria | 1960 |
| Tuerkis | Germany | 2004 |
| Unterfraenkischer Landweizen | Germany | 1950 |
| Vilmorin 27 | France | 1928 |
| Virgo | Sweden | 1968 |
| Weibulls 18609 | Sweden | 1972 |
| Werla | Germany | 1950 |
| Wiwa | Germany | NA |
| Xerxes | Germany | 2013 |
| Xi19 | Great Britain | 2002 |

**Table S2** Marker-trait associations surpassing the explorative significance threshold P <0.001 (3) indicating putative quantitative trait loci (QTL) for the eight ATI proteins and total ATI

| **Trait** | **Marker** | **Chr.** | **Gen Pos (cM)** | **Phy Pos (bp)** | **LOD** | ***P*_G_** | **α-Effect** |
| --- | --- | --- | --- | --- | --- | --- | --- |
| **ATI 0.28** |  |  |  |  |  |  |  |
|  | 1108014D | 7A | 259.0 | 699796602 | 3.72 | 2.21 | 1.66 |
|  | 1106155D | 6B | 6.5 | 2094331 | 13.71 | 68.73 | 3.30 |
|  | 1211376D* | 6B | 6.9 | - | 8.38 | 1.41 | -2.86 |
|  | 1111022D* | 6B | 8.4 | 6042987 | 3.38 | 1.70 | -2.20 |
|  | 1075928D | 6B | 12.4 | - | 4.47 | 1.48 | 2.10 |
| **ATI 0.19** | |  |  |  |  |  |  |
|  | 1109237D | 1B | 16.6 | 1972615 | 3.18 | 17.93 | -4.47 |
|  | 997799D | 1B | 61.6 | 10086260 | 3.10 | 1.95 | -5.01 |
|  | 1863254S* | 3B | 87.9 | - | 3.11 | 5.93 | 9.44 |
|  | 1050374S* | 3B | 160.6 | 710587539 | 3.41 | 25.96 | 9.62 |
|  | 1279364D | 3B | 275.0 | 810282553 | 3.04 | 12.47 | -3.36 |
| **ATI 0.19-like** |  |  |  |  |  |  |  |
|  | 2275974D | 3B | 93.8 | 78749798 | 10.45 | 70.55 | -2.67 |
|  | 1123753S | 3B | 93.8 | 78136767 | 7.97 | 7.58 | -1.91 |
|  | 2250389D* | 3B | 93.8 | 335124804 | 5.87 | 1.06 | 2.06 |
|  | 1115028D | 3B | 113.1 | 468749819 | 3.27 | 10.22 | -1.27 |
|  | 3027580D | 5B | 153.6 | 564310048 | 3.89 | 3.54 | 1.45 |
|  | 2279020D | 6B | 27.7 | 29989810 | 3.07 | 2.47 | 0.79 |
|  | 1058060D | 7B | 202.4 | 343362064 | 3.53 | 7.42 | 0.93 |
|  | 1130165D* | 2D | 274.4 | 630631214 | 3.26 | 2.19 | 0.85 |
|  | 1106297D | 7D | 148.4 | 115020555 | 3.02 | 4.65 | -0.61 |
| **ATI CM1** |  |  |  |  |  |  |  |
|  | 1767073S* | 1A | 125.5 | 29994152 | 3.20 | 14.87 | 1.03 |
|  | 1299160D | 6A | 189.0 | 610285943 | 3.06 | 9.20 | -0.90 |
|  | 3960850D* | 1B | 169.0 | 156688612 | 3.53 | 27.61 | -1.00 |
|  | 1091381S* | 4B | 66.6 | 383656519 | 3.17 | 21.46 | -0.55 |
| **ATI CM2** |  |  |  |  |  |  |  |
|  | 3960850D* | 1B | 169.0 | 156688612 | 3.66 | 13.07 | -0.67 |
|  | 1283844D* | 7B | 44.5 | 83795937 | 4.85 | 30.51 | 0.58 |
|  | 3384625D* | 7B | 58.4 | 600924343 | 4.57 | 1.31 | -0.55 |
|  | 2326882D | 7B | 58.4 | 74482621 | 4.23 | 1.26 | 0.55 |
|  | 1142012D* | 7B | 83.6 | 148945778 | 3.27 | 5.48 | -0.53 |
|  | 2252729D | 1D | 103.9 | 235161835 | 3.28 | 13.55 | -0.34 |
| **ATI CM3** |  |  |  |  |  |  |  |
|  | 1131064D | 1A | 142.2 | 287707863 | 3.47 | 13.24 | 0.9 |
|  | 1095173D* | 5A | 164.3 | 583031356 | 3.80 | 23.43 | -0.75 |
|  | 1089167D | 5A | 164.3 | 582654179 | 3.49 | 1.45 | -0.78 |
|  | 3955268D* | 6A | 168.3 | 600392875 | 3.11 | 8.66 | 1.03 |
|  | 1100926D | 3B | 102.9 | 171597291 | 3.09 | 3.19 | -1.35 |
|  | 1134744D | 4B | 63.9 | 226827187 | 3.53 | 3.39 | -0.58 |
|  | 1091381S* | 4B | 66.6 | 383656519 | 3.68 | 21.67 | -0.87 |
|  | 2289413D | 7B | 88.3 | 240398218 | 3.10 | 5.85 | -0.6 |
|  | 1123562D | 1D | 131.7 | 373654626 | 3.13 | 4.05 | -0.59 |
|  | 1061909D | 6D | 0.7 | 1619257 | 3.38 | 4.74 | -0.91 |
|  | 1120428D | 6D | 2.0 | - | 3.41 | 11.86 | 1.27 |
|  | 3941872D* | 6D | 196.2 | 467815692 | 3.32 | 8.9 | -1.45 |
| **ATI CM16** |  |  |  |  |  |  |  |
|  | 1717322D* | 1A | 120.1 | 348028262 | 3.16 | 1.87 | 1.52 |
|  | 987095S | 1A | 125.5 | 51346989 | 3.42 | 8.77 | 0.99 |
|  | 1131064D | 1A | 142.2 | 287707863 | 3.18 | 5.49 | 0.84 |
|  | 1017299S* | 5A | 164.3 | 576949459 | 3.69 | 16.9 | -1.6 |
|  | 1093432S | 1B | 186.8 | 458325636 | 3.26 | 6.63 | 1.26 |
|  | 3021168D | 2B | 62.6 | 48288422 | 3.16 | 3.68 | 0.83 |
|  | 1862786S | 2B | 197.6 | 782126877 | 3.15 | 5.88 | -1.16 |
|  | 1100926D | 3B | 102.9 | 171597291 | 3.09 | 3.66 | -1.43 |
|  | 1143991D* | 6B | 5.4 | - | 3.49 | 17.01 | 1.06 |
|  | 1204418D | 7B | 83.6 | 148945775 | 3.14 | 2.18 | -1.08 |
|  | 1108249D* | 7B | 95.2 | 531222743 | 3.46 | 18.49 | -1.61 |
|  | 3385231D* | 7B | 217.2 | 709321814 | 4.11 | 28.77 | -0.82 |
|  | 1008216D | 7B | 217.2 | 711156950 | 3.34 | 1.37 | -0.78 |
| **ATI CM17** |  |  |  |  |  |  |  |
|  | 3960850D* | 1B | 169.0 | 156688612 | 3.18 | 27.83 | -1.35 |
|  | 3955247S* | 3B | 102.9 | 172016539 | 3.05 | 5.69 | 1.35 |
|  | 1091381S* | 4B | 66.6 | 383656519 | 4.34 | 23.60 | -0.88 |
|  | 2300559D | 7D | 79.3 | 40594313 | 3.11 | 11.35 | -0.8 |
| **Total ATI** |  |  |  |  |  |  |  |
|  | 1090273D | 1A | 120.1 | 49355051 | 3.02 | 11.97 | -8.17 |
|  | 3955268D* | 6A | 168.3 | 600392875 | 3.25 | 15.97 | 9.72 |
|  | 1089810D | 1B | 104.0 | 1206329 | 3.02 | 15.4 | -10.39 |
|  | 1248458D | 2B | 202.5 | 797080098 | 3.27 | 13.74 | -6.62 |
|  | 3955247S* | 3B | 102.9 | 172016539 | 3.30 | 27.08 | 13.17 |
|  | 1100926D | 3B | 102.9 | 171597291 | 3.27 | 1.03 | -12.70 |
|  | 1279364D | 3B | 275.0 | 810282553 | 3.36 | 25.06 | -5.72 |
|  | 3222133S | 3B | 104.1 | 305093061 | 3.16 | 3.02 | 13.06 |
|  | 1197486D | 7B | 0 | 1134363 | 3.01 | 4.52 | -7.10 |
|  | 1096867D | 2D | 47.5 | 28561530 | 3.09 | 13.62 | -7.58 |

Chr. Chromosome, Gen Pos chromosome position in cM, Phy Pos sequence start position in base pairs according to the bread wheat reference genome (IWGSC RefSeq v1.0), *P*_G_ proportion of genotypic variance explained by the QTL in percent, and allele substitution (α) effect.

* Unmapped marker placed on the map based on the position of mapped markers in high linkage disequilibrium with it.

**Table S3** 1081 genes extracted from the identified chromosomal regions of the significant QTL explaining more than 10% of genotypic variance after fine mapping. Chr. Chromosome, ID Identifier, GO gene ontology. See Excel Table

**Fig. S1** Boxplots showing the amount of different ATI proteins in the wheat cultivars depending on the country, where the wheat cultivars were registered. Numbers in the plot indicate the mean values and the number of cultivars in each group. (DE, Germany; FRA, France; GBR, Great Britain; BE+NL, Belgium + Netherland; IT+CH, Italy + Switzerland; CEEU, Central-Eastern European countries: Poland, Austria, Hungary, ex-Yugoslavia; NEU, Northern European countries: Sweden and Denmark).

**Fig. S2** Manhattan plots showing significant marker-trait association for six ATIs and total ATI content at explorative significance threshold *P* < 0.001 (dashed line). The x-axis shows the DArTseq markers on 21 chromosomes based on the genetic map positions (cM) and the y-axis shows the P-values on a −log_10_ scale

**
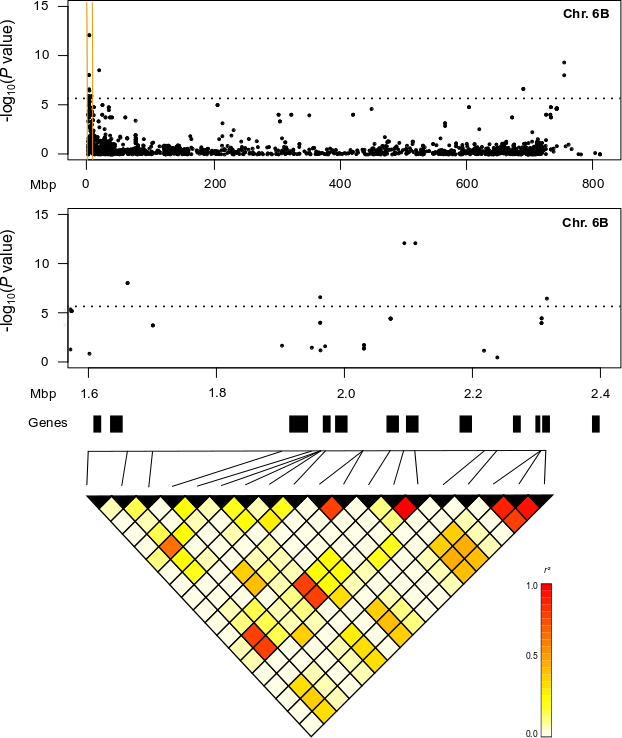
**

**Fig. S3** Fine-mapping of the major QTL (1106155D) for ATI 0.28 on chromosome 6B. Genes detected in the target region 1.6-2.4 Mbp on chromosome 6B are marked by black boxes. Linkage disequilibrium (*r²*) among all the markers in the region 1.6-2.4 Mbp is shown in the heatmap

**Fig. S4** Cross-validated prediction ability for eight ATI proteins and total ATI based on the identified QTL explaining more than 10% of the genotypic variance (MAS), genome-wide prediction with ridge-regression BLUP (RR-BLUP), or based on weighted ridge regression BLUP (wRR-BLUP) incorporating the QTL explaining more than 10 % of the genotypic variance as fixed effects in the model
